# Notice of withdrawal of the article “Pediatric case of trichilemmal cyst arising on the face”

**DOI:** 10.1016/j.abd.2024.07.001

**Published:** 2024-10-22

Withdrawal notice to <Pediatric case of trichilemmal cyst arising on the face>

<[An Bras Dermatol. 2024;99(2):297-299]>

<Mai Endo∗, Toshiyuki Yamamoto>

<Department of Dermatology, Fukushima Medical University, Fukushima, Japan>

The Publisher regrets that this article is an accidental duplication of an article that has already been published in <An Bras Dermatol. 2024;99(1):130-131>, http://dx.doi.org/<10.1016/j.abd.2022.05.012>. The duplicate article has therefore been withdrawn.

The full Elsevier Policy on Article Withdrawal can be found at https://www.elsevier.com/about/policies/article-withdrawal.

